# Real-time three-dimensional systolic dyssynchrony index predicts pacing-induced left ventricular dysfunction and cardiomyopathy

**DOI:** 10.1007/s00392-026-02921-7

**Published:** 2026-04-27

**Authors:** Moustafa Dawood, Hoda Abdelgawad, Aly Aboelhoda, Moataz Zaki

**Affiliations:** 1 Faculty of Medicine, Alexandria University, Alexandria, Egypt; 2 King’s College Hospital NHS Trust, London, UK; 3 Medical Research Institute, Alexandria University, Alexandria, Egypt

**Keywords:** Pacing-induced cardiomyopathy, Systolic dyssynchrony index, 3D echocardiography, Right ventricular pacing, Global longitudinal strain

## Abstract

**Background:**

Right ventricular pacing leads to left ventricular (LV) dyssynchrony, which is a critical factor in the pathophysiology of pacing-induced LV dysfunction (PIVD) and cardiomyopathy (PICM).

**Objectives:**

This study aimed to determine the incidence of PIVD and PICM and evaluate the prognostic value of the real-time three-dimensional echocardiography (RT3DE)-derived systolic dyssynchrony index (SDI), measured shortly after pacemaker implantation, in predicting their development.

**Methods:**

In this prospective observational study, 108 patients undergoing permanent RV pacing. Only 60 patients with preserved baseline LV ejection fraction (LVEF ≥ 50%) were enrolled. Comprehensive echocardiography, including global longitudinal strain (GLS) and RT3DE for SDI (Tmsv 16-SD) calculation, was performed at baseline, 7–10 days, and 6 months post-implantation. Patients were classified at 6 months as preserved (LVEF reduction ≤ 10%, final LVEF ≥ 50%), PIVD (LVEF reduction > 10%, final LVEF ≥ 50%), or PICM (LVEF reduction > 10%, final LVEF < 50%).

**Results:**

The patients’ mean age was 62.4 ± 16.3 years, 32 patients were males (53.3%). Mean follow-up was 6.53 ± 0.75 months. Twenty-five patients (41.7%) developed LV impairment; of them, 16 (85%) patients had PIVD, and nine (15%) patients had PICM. While baseline characteristics were similar, the dysfunction group exhibited significantly higher SDI and worse GLS as early as 7–10 days post-pacing. At 6 months, there were significant differences between the preserved group (35 patients) and impaired LV group regarding LV EF (63.6 ± 5 vs. 49.8 ± 11.6, *p* < 0.001), GLS (− 15.6 ± 3 vs. − 10.9 ± 3.8, *p* < 0.001), SDI% (2.4 ± 1.2 vs. − 7.9 ± 5.9, *p* < 0.001), and excursion (5.8 ± 2.23 vs. 3.2 ± 2.1, *p* < 0.001). In multivariate analysis, both SDI (odds ratio [OR], 5.38; 95% CI, 1.56–18.52; *p* = 0.008) and GLS (OR, 1.39; 95% CI, 1.02–1.90; *p* = 0.040) at 7–10 days were independent predictors of 6-month dysfunction. Receiver operating characteristic analysis showed that an SDI > 1.49% predicted dysfunction with an area under the curve of 0.843 (84% sensitivity, 71% specificity). A clear gradient of worsening SDI and GLS was observed from Preserved to PIVD to PICM.

**Conclusions:**

Mechanical dyssynchrony quantified by RT3DE-derived SDI is a powerful, independent predictor of pacing-induced LV dysfunction, identifiable within the first week after implantation. This early measurement offers a critical opportunity for risk stratification and guided intervention to prevent the progression to overt cardiomyopathy.

**Graphical Abstract:**

**Figure:**
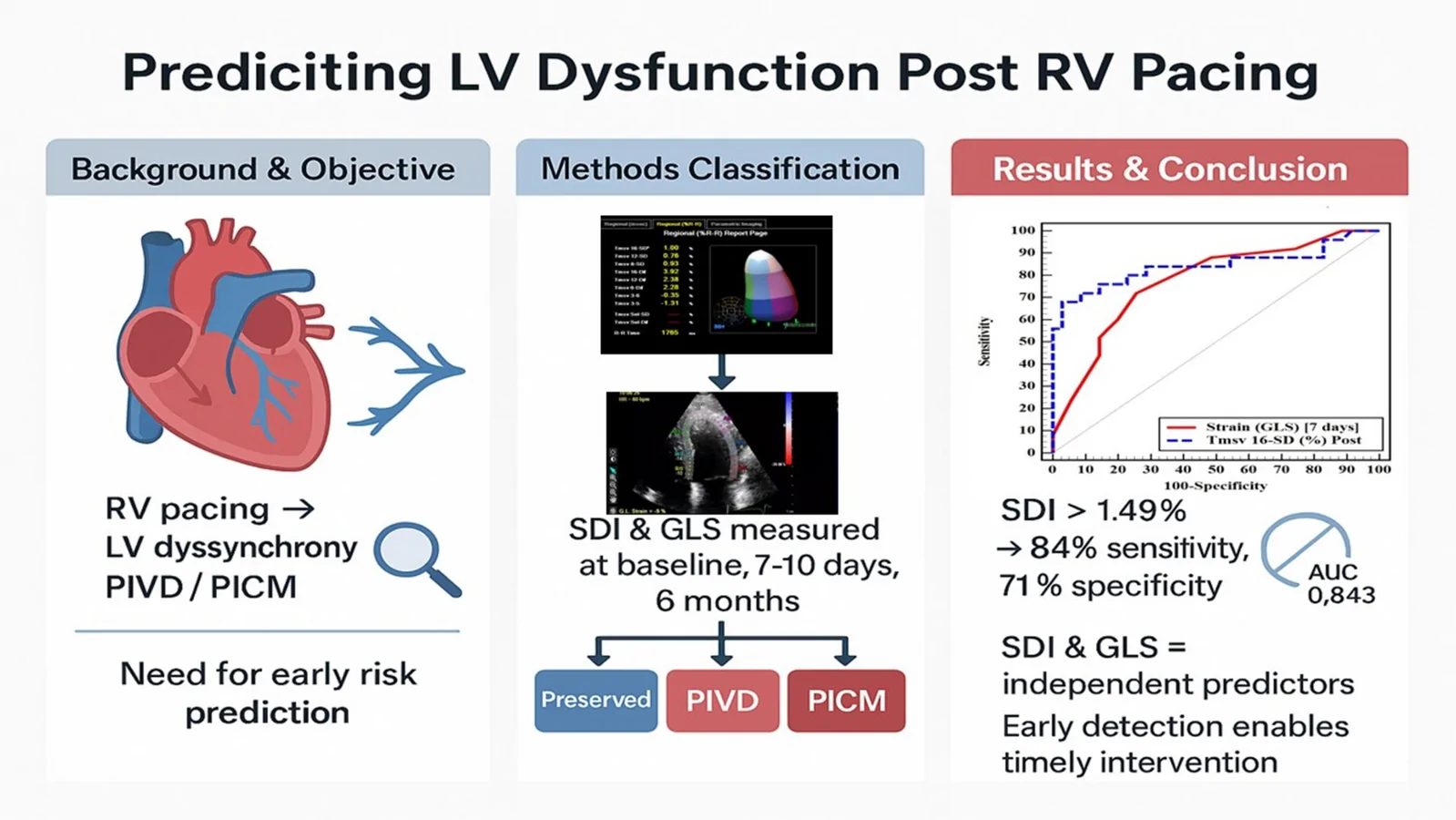

**Supplementary Information:**

The online version contains supplementary material available at https://doi.org/10.1007/s00392-026-02921-7.

## Introduction

Cardiac pacing is a life-saving therapy for patients with bradyarrhythmias. However, chronic right ventricular (RV) pacing may induce left ventricular (LV) dysfunction [1]. By creating an abnormal electrical activation sequence that mimics left bundle branch block, RV pacing leads to mechanical dyssynchrony, characterized by paradoxical septal wall motion, discoordinate contraction, and reduced pumping efficiency [2, 3]. This iatrogenic dyssynchrony can initiate a cascade of adverse LV remodeling, ultimately culminating in heart failure, a condition broadly termed pacing-induced cardiac dysfunction [4, 5].

The spectrum of this disease is now recognized to include distinct stages of severity. Pacing-induced ventricular dysfunction (PIVD) represents the more common, subclinical stage, typically defined as a greater than 10% reduction in LV ejection fraction (LVEF) from baseline while the absolute LVEF remains preserved (≥ 50%) [6]. In a proportion of patients, this dysfunction progresses to the more severe stage of pacing-induced cardiomyopathy (PICM), defined as a decline in LVEF to below 50% (often with an absolute reduction of > 10%) in the absence of other identifiable causes [7]. The clinical significance of both entities is profound, as they are associated with a higher risk of heart failure hospitalizations, atrial fibrillation, and increased mortality [8, 9]. The reported incidence varies widely, influenced by the definition used [7], patient population, and follow-up duration. Systematic reviews suggest that while overt PICM affects approximately 10–15% of patients, the incidence of the broader category of significant dysfunction (PIVD) can be much higher, exceeding 30–40% in high-risk cohorts, such as those with a very high RV pacing burden [10].

This variability in incidence underscores a critical problem: the identification of patients at risk remains imprecise. Traditional risk factors like a high RV pacing burden, pre-existing heart disease, and a wide-paced QRS complex lack sufficient predictive accuracy for individual patient management. There is a growing consensus that the substrate for pacing-induced dysfunction is mechanical, not purely electrical. Consequently, direct quantification of mechanical dyssynchrony may offer superior predictive value [11]. This has led to the exploration of more sensitive markers, such as a relative reduction in global longitudinal strain (GLS) by speckle-tracking echocardiography, which can detect subtle myocardial injury before a fall in LVEF becomes apparent [12].

The systolic dyssynchrony index (SDI), derived from real-time three-dimensional echocardiography (3DE), has emerged as a robust tool for assessing global LV mechanical dyssynchrony [13]. Unlike two-dimensional techniques, 3DE captures the entire LV volume in a single heartbeat, and the SDI is calculated as the standard deviation of the time to minimum systolic volume for all LV segments, providing a comprehensive measure of temporal contraction disparity [14]. While its utility in predicting response to cardiac resynchronization therapy is established [15], its role in predicting the onset of dysfunction after RV pacing is less defined, particularly its ability to distinguish the very early stages of PIVD.

Therefore, this study aimed, first, to determine the incidence of both PIVD and PICM in a contemporary cohort of patients with a high burden of RV pacing and, second, to evaluate the role of the real-time three-dimensional SDI, measured shortly after implantation, in predicting the subsequent development of significant pacing-induced LV dysfunction.

## Methods

### Study design and population

This prospective observational study was conducted at Alexandria Main University Hospitals. Between April 2025 and November 2025, from 108 patients scheduled for permanent pacemaker implantation with an anticipated high burden of RV pacing, only 60 patients met the inclusion criteria and reached follow-up. All participants provided written informed consent, and the study was approved by the local institutional ethics committee.

Inclusion criteria were (1) conventional clinical indications for permanent pacemaker implantation and (2) a preserved baseline LVEF (≥ 50%) confirmed by pre-implantation 3D echocardiography. Key exclusion criteria were designed to isolate the effect of pacing on LV function: (1) poor echocardiographic windows precluding accurate 3DE analysis, (2) baseline LVEF < 50%, (3) significant valvular heart disease (moderate or severe stenosis or regurgitation), (4) history of acute coronary syndrome or coronary revascularization within 3 months before enrollment or up to 6 months after device insertion, (5) pacing indication due to sick sinus syndrome or slow atrial fibrillation (to minimize inclusion of patients with potentially lower pacing burdens), and (6) RV pacing percentage of less than 20% during follow-up, confirmed by device interrogation.

### Echocardiographic acquisition and analysis

A comprehensive echocardiographic examination was performed at three time points: within 24hour prior to implantation (baseline), 7–10 days post-implantation, and at the 6-month follow-up. All studies were performed using the Philips Medical iE33 Echocardiography system equipped with an X5-1 xMATRIX transthoracic probe.

#### Two-dimensional echocardiography

Standard ECG-gated apical two-, three-, and four-chamber views were acquired during a brief breath-hold. LVEF was calculated using the biplane Simpson’s method. For speckle-tracking echocardiography, images were stored in cine-loop format and analyzed offline using a semi-automated software package (QLAB, Philips) to obtain global longitudinal strain (GLS). GLS was calculated as the average peak systolic strain from the apical views.

#### Three-dimensional echocardiography

For the assessment of LV volumes and mechanical dyssynchrony, real-time three-dimensional echocardiography (RT3DE) was performed. Full-volume datasets were acquired from the apical window. The system obtained a pyramidal volume (approximately 30° × 30°) over 6 consecutive cardiac cycles during a single breath-hold to ensure an adequate volume rate (targeting 20–35 volumes per second) and to avoid stitch artifacts. The resulting dynamic 3D dataset was reviewed immediately to ensure the entire LV cavity was included and that no significant translation artifacts were present.

The 3D datasets were transferred to a dedicated workstation for offline analysis using QLAB 3DQ Advanced software (Philips Medical Systems). The analysis protocol was as follows:

1. LV volume and EF analysis: 

The apical four-chamber and two-chamber views and a parasternal short-axis view were automatically displayed. After initial manual identification of five reference points (four at the mitral annulus and one at the apex) on the end-diastolic and end-systolic frames, a pre-configured ellipse was fitted to the endocardial border. The software then semi-automatically traced the endocardial border throughout the cardiac cycle. Manual adjustments were performed when necessary to ensure optimal tracking. The software subsequently generated a dynamic 3D model of the LV and automatically calculated end-diastolic volume (EDV), end-systolic volume (ESV), stroke volume (SV), and LVEF.

2. Assessment of LV dyssynchrony (systolic dyssynchrony index, SDI)

Using the same 3D model, the software automatically divided the LV into 16 standard wedge-shaped segments. For each segment, a time-volume curve was generated, and the time taken to reach the minimum systolic volume (Tmsv) was calculated. The systolic dyssynchrony index (SDI) was automatically derived as the standard deviation of the Tmsv for all 16 segments (Tmsv 16-SD), expressed as a percentage of the cardiac cycle, providing a quantitative measure of global LV mechanical dyssynchrony[14]. A regional assessment for 12-segment (Tmsv12-SD) and 6-segment (Tmsv6-SD) LV models is available. In addition, the maximum time difference between the onset of QRS and the moment of reaching the minimum regional systolic volume was determined for each of the models, with an indication of the number of segments included in the model (Tmsv16-Dif, Tmsv12-Dif, Tmsv6-Dif).

To analyze the segmental contractility (regional movement of the endocardium toward the central axis, drawn from the basal sections to the apex and calculated using the time-volume curves), the parameters obtained from the RT3DE analysis were used to draw two bull’s-eye color charts. The upper one expresses Tmsv for each segment, where green codes for normal time to reach the minimum regional volume, blue codes for early-activated areas, and red codes for late-activated areas. The other chart displays the excursion (direction and extent of displacement) for each segment toward or away from the LV central axis. This is expressed as the average excursion value (Excursion Avg), its standard deviation (Excursion SD), the maximum (Excursion Max), the minimum (Excursion Min) and the threshold values (Excursion Threshold), measured in mm. It is expressed using color coding (the blue color codes movement toward the central axis, red codes movement away from it, and black codes the lack of movement).

For a comprehensive and concise evaluation of global LV mechanics, the results of the 16-segment model (Tmsv 16-SD) and average excursion values were adopted. The remaining values were deposited as additional data for viewing.

### Device implantation

All devices were implanted by experienced electrophysiologists via the subclavian vein. The ventricular lead was positioned under fluoroscopic guidance. The specific site of ventricular lead placement, whether the right ventricular apex (RVA) or the septum (RVS), was recorded for each patient. The atrial lead was positioned in the right atrial appendage. All patients received either a single-chamber (VVI) or a dual-chamber (DDD) pacemaker. The devices were programmed in DDD or VVI mode with a lower rate limit of 60 beats per minute, and the atrioventricular (AV) delay was optimized echocardiographically to maximize trans-mitral inflow duration.

### Outcome definitions and follow-up

Patients were followed twice at 7–10 days and at 6 months post-implantation. Echocardiographic assessment was performed as described before in addition to device interrogation. The first follow-up was postponed up to 10 days instead of 24 h post-implantation because it was difficult to obtain standard views while the dressing was still in place. We preferred to perform imaging acquisition after dressing removal at 7–10 days. Based on the 6-month echocardiogram, patients were categorized into three groups:

- PICM Group: Defined as a relative reduction in LVEF > 10% from baseline resulting in an absolute LVEF < 50%.
- PIVD Group: Defined as a relative reduction in LVEF > 10% from baseline with the final LVEF remaining ≥ 50%.
- Preserved LV Function Group: Defined as no significant change in LVEF (≤ 10% reduction from baseline).

For the primary analysis of predicting any significant pacing-induced dysfunction, the PIVD and PICM groups were combined into one combined “Dysfunction Group” and compared against the Preserved Function group.

Device interrogation was performed at the 7–10-day and 6-month visits to confirm sensing/pacing parameters and to document the percentage of RV pacing.

### Statistical analysis

Data were analyzed using IBM SPSS software version 20.0 (Armonk, NY: IBM Corp, released 2011). All continuous variables were tested for normality using the Shapiro–Wilk test and visual inspection of Q-Q plots. Normally distributed continuous variables are presented as mean ± standard deviation, while non-normally distributed variables are presented as median (interquartile range). Categorical variables are presented as numbers and percentages.

#### Between-group comparisons

Comparisons between the Preserved and Dysfunction groups were performed using the independent Student’s *t*-test for normally distributed continuous variables and the Mann–Whitney *U* test for non-normally distributed continuous variables. Categorical variables were compared using the Chi-square test or Fisher’s exact test, as appropriate. For the three-subgroup analyses (Preserved vs. PIVD vs. PICM), one-way ANOVA with post hoc Tukey HSD test was used for normally distributed variables, and the Kruskal–Wallis test with post hoc Dunn’s test for multiple comparisons was used for non-normally distributed variables.

#### Repeated measures analysis

Serial changes in echocardiographic parameters across the three time points (baseline, 7–10 days, 6 months) were analyzed using linear mixed-effects models with an unstructured covariance matrix to account for within-subject correlation. Time, group, and time-by-group interaction were included as fixed factors. Post hoc pairwise comparisons with Bonferroni correction for multiple comparisons were performed for significant interaction terms to identify specific time points where groups differed.

#### Correlation analysis

Associations between Tmsv 16-SD and other continuous variables (including ESV, EF%, QRS width, ventricular pacing percentage, GLS, and Average Excursion) were assessed using Spearman’s rank correlation coefficient (*r*_s_). Spearman’s method was selected a priori for several reasons. First, Shapiro–Wilk testing demonstrated that several key variables, including Tmsv 16-SD and ventricular pacing percentage, were not normally distributed, violating the assumptions required for Pearson’s correlation. Second, the modest sample sizes within subgroups (*n* = 35 in the Preserved group, *n* = 25 in the Dysfunction group) precluded the reliable assumption of bivariate normality, which is necessary for parametric correlation methods. Third, Spearman’s rank correlation is robust to the presence of outliers and does not require linearity assumptions, making it particularly suitable for exploratory analyses in modest-sized clinical cohorts where the underlying relationship between variables may not be strictly linear.

For categorical variables (device type, lead site), differences in Tmsv 16-SD between categories were assessed using the Mann–Whitney *U* test (for two categories) or Kruskal–Wallis test (for three or more categories), as SDI was non-normally distributed. The small number of patients in some lead-site subgroups (e.g., RVFW, *n* = 1 in the Dysfunction group) is explicitly noted as a limitation.

#### Regression analysis

Univariate logistic regression analysis was performed to identify predictors of LV dysfunction at 6 months. Variables with *p* < 0.10 in univariate analysis were entered into a multivariate forward-stepwise logistic regression model (entry criterion *p* ≤ 0.05, removal criterion *p* ≥ 0.10). The Hosmer–Lemeshow test was used to assess goodness-of-fit. Results are reported as odds ratios (OR) with 95% confidence intervals (CI).

#### Predictive performance

The prognostic performance of SDI and GLS to predict LV dysfunction was assessed using receiver operating characteristic (ROC) curve analysis. The optimal cutoff value was determined by the Youden index (sensitivity + specificity − 1). Sensitivity, specificity, positive predictive value (PPV), and negative predictive value (NPV) were calculated. Area under the curve (AUC) values were compared using DeLong’s method for paired ROC curves.

## Results

### Patient characteristics and incidence of PIVD and PICMP

Initially, 108 patients were assessed for eligibility. After applying the inclusion and exclusion criteria, 48 patients were excluded for various reasons detailed in Supplementary Fig. A. Consequently, the final study cohort consisted of 60 patients who completed the 6-month follow-up and were included in the analysis. During the 6-month follow-up, 25 patients (41.7%) developed significant LV dysfunction. Of these, 16 patients (26.7% of the total cohort) met the criteria for PIVD, while 9 patients (15.0% of the total cohort) progressed to PICMP. The remaining 35 patients (58.3%) maintained preserved LV function. The baseline clinical and demographic characteristics of the entire cohort and the two compared groups (Preserved vs. Combined Dysfunction) are summarized in (Supplementary Table A). The groups were well-matched, with no significant differences in age, sex, comorbidities, device type, baseline/paced QRS duration/axis, ventricular lead position, or RV pacing burden. When comparing the three subgroups (Preserved, PIVD, PICM), no significant differences are found in these baseline parameters (Table 1).

**Table 1:** Comparison between the three studied subgroups (preserved, PIVD, PICM) according to according to demographic, ECG, and device characteristics

	Preserved (*n* = 35)	PIVD (*n* = 16)	PICM (*n* = 9)	*p*
Sex				
Male	19 (54.3%)	6 (37.5%)	7 (77.8%)	^MC^*p* = 0.163
Female	16 (45.7%)	10 (62.5%)	2 (22.2%)	
Age (years)				
Mean ± SD	63.11 ± 18.99	60.63 ± 13.01	63.0 ± 10.48	0.878
DM	10 (28.6%)	2 (12.5%)	3 (33.3%)	^MC^*p* = 0.412
HTN	15 (42.9%)	5 (31.3%)	4 (44.4%)	0.703
IHD	0 (0.0%)	0 (0.0%)	1 (11.1%)	^MC^*p* = 0.153
Heart rate				
Pre				
Mean ± SD	43.20 ± 10.26	42.69 ± 6.88	36.44 ± 3.81	0.099
Post (7–10 days)				
Mean ± SD	70.83 ± 12.74	67.44 ± 9.85	73.78 ± 16.82	0.650
Follow up (6 months)				
Mean ± SD		66.63 ± 8.59	72.56 ± 14.68	0.575
Native QRS width				
Mean ± SD	115.1 ± 31.93	124.4 ± 35.26	130.0 ± 37.42	0.414
Paced QRS width				
Mean ± SD	153.9 ± 22.03	154.1 ± 21.07	148.9 ± 28.48	0.830
Paced QRS axis				
Normal	6 (17.1%)	2 (12.5%)	1 (11.1%)	^MC^*p* = 0.824
Right	1 (2.9%)	2 (12.5%)	0 (0.0%)	
Ex. right	1 (2.9%)	0 (0.0%)	0 (0.0%)	
Left	27 (77.1%)	12 (75.0%)	8 (88.9%)	
Device type				
VVI	21 (60.0%)	8 (50.0%)	3 (33.3%)	^MC^*p* = 0.388
DDD	14 (40.0%)	8 (50.0%)	6 (66.7%)	
Lead site				
RVA	27 (77.1%)	14 (87.5%)	7 (77.8%)	^MC^*p* = 0.752
RVS	4 (11.4%)	2 (12.5%)	1 (11.1%)	
RVFW	4 (11.4%)	0 (0.0%)	1 (11.1%)	
V. pacing (%) [7-10 days]				
Mean ± SD	77.81 ± 27.50	84.27 ± 32.08	94.78 ± 9.44	0.239
V. pacing (%) [ 6 months]				
Mean ± SD	81.31 ± 26.46	97.19 ± 6.92	97.44 ± 6.62	0.183

*p p*-value for comparing between the three studied groups

^*^Statistically significant at *p* ≤ 0.05

For examples of a case of PIVD and a case of PICM, by 3D RT SDI and LV GLS, kindly read the Supplementary Material.

### Temporal changes in left ventricular volumes and function

The serial changes in 3D echocardiographic parameters are presented in (Supplementary Table B). At baseline (pre-implantation), there were no significant differences between the Preserved and Dysfunction groups in end-diastolic volume (EDV), end-systolic volume (ESV), ejection fraction (EF%), stroke volume (SV), or cardiac output (COP).

Following pacemaker implantation, significant differences emerged. At the 7–10-day post-operative assessment, the Dysfunction group already demonstrated a significantly larger ESV (37.52 ± 9.96 mL vs. 28.31 ± 11.51 mL, *p* = 0.002) and a lower EF% (59.40 ± 9.60% vs. 65.97 ± 6.72%, *p* = 0.005) compared to the Preserved group. These differences became more pronounced at the 6-month follow-up. The Dysfunction group showed a marked increase in ESV (47.24 ± 23.26 mL vs. 33.60 ± 10.87 mL, *p* = 0.004) and a substantial decline in EF% (49.84 ± 11.57% vs. 63.60 ± 5.04%, *p* < 0.001). Cardiac output at 6 months was also significantly lower in the Dysfunction group (3.21 ± 0.89 L/min vs. 3.81 ± 1.22 L/min, *p* = 0.041).

The analysis of the three subgroups (Table 2) reveals a clear gradient of worsening function from Preserved to PIVD to PICM. At 6 months, the PICM subgroup had the highest ESV (63.33 ± 32.0 mL) and the lowest EF% (37.11 ± 9.82%), with all pairwise comparisons between subgroups being statistically significant (*p* < 0.05).

**Table 2:** Comparison between the three studied subgroups (Preserved, PIVD, PICM) according to according to 3D echo–derived LV volumes, ejection fraction, stroke volume, and cardiac output

		Preserved (*n* = 35)	PIVD (*n* = 16)	PICM (*n* = 9)	*p*	Sig. bet. grps
EDV	Pre					
	Mean ± SD	103.2 ± 27.75	103.9 ± 16.74	110.4 ± 21.35	0.725	–
	Post (7–10 days)					
	Mean ± SD	84.86 ± 24.12	93.94 ± 17.69	92.56 ± 20.43	0.336	–
	Follow–up (6 months)					
	Mean ± SD	88.69 ± 24.50	89.56 ± 21.21	104.4 ± 33.99	0.245	–
ESV	Pre					
	Mean ± SD	31.86 ± 12.05	27.69 ± 6.44	36.44 ± 10.51	0.142	–
	Post (7–10 days)					*p*_1_ = 0.213
	Mean ± SD	28.31 ± 11.51	33.69 ± 9.74	44.33 ± 6.18	0.001^*^	*p*_2_ < 0.001^*^ *p*_3_ = 0.046^*^
	Follow–up (6 months)					*p*_1_ = 0.584
	Mean ± SD	33.60 ± 10.87	38.19 ± 8.75	63.33 ± 32.0	< 0.001^*^	*p*_2_ < 0.001^*^ *p*_3_ = 0.001^*^
EF%	Pre					
	Mean ± SD	69.40 ± 6.50	73.0 ± 4.82	66.56 ± 8.93	0.053	–
	Post (7–10 days)					*p*_1_ = 0.885
	Mean ± SD	65.97 ± 6.72	65.06 ± 5.80	49.33 ± 5.96	< 0.001^*^	*p*_2_ < 0.001^*^ *p*_3_ = 0.001^*^
	Follow–up (6 months)					*p*_1_ = 0.001^*^
	Mean ± SD	63.60 ± 5.04	57.0 ± 3.29	37.11 ± 9.82	< 0.001^*^	*p*_2_ < 0.001^*^ *p*_3_ = 0.001^*^
SV	Pre					
	Mean ± SD	71.40 ± 19.55	74.75 ± 13.17	74.0 ± 22.69	0.816	–
	Post (7–10 days)					
	Mean ± SD	56.43 ± 16.81	60.31 ± 11.13	48.22 ± 15.87	0.177	–
	Follow–up (6 months)					
	Mean ± SD	55.09 ± 16.18	52.13 ± 14.22	41.11 ± 11.34	0.054	–
COP	Pre					
	Mean ± SD	3.11 ± 1.08	3.24 ± 0.81	2.62 ± 0.59	0.285	–
	Post (7–10 days)					
	Mean ± SD	4.05 ± 1.18	4.09 ± 1.06	3.47 ± 1.03	0.350	–
	Follow–up (6 months)					
	Mean ± SD	3.81 ± 1.22	3.41 ± 0.97	2.86 ± 0.64	0.061	–

*F*: *F* for One way ANOVA test; pairwise comparison between each 2 groups was done using post hoc test (Tukey)

*p p-*value for comparing between the three studied subgroups, *p*_1_ *p-*value for comparing between preserved and PIVD, *p*_2_ *p-*value for comparing between preserved and PICM, *p*_3_ *p-*value for comparing between PIVD and PICM

^*^Statistically significant at *p* ≤ 0.05

### Myocardial deformation and systolic dyssynchrony

Global longitudinal strain (GLS) and systolic dyssynchrony index (Tmsv 16-SD) results are shown in (Supplementary table C). Baseline GLS was comparable between the groups. However, after just 7–10 days post-implantation, GLS was significantly more impaired in the Dysfunction group (− 13.2 ± 3.22%) than in the Preserved group (− 16.7 ± 3.18%, *p* < 0.001). The percentage change in GLS from baseline was substantially greater in the Dysfunction group (30.24 ± 10.40% vs. 10.46 ± 8.42%, *p* < 0.001), indicating rapid deterioration of myocardial function. This trend continued at 6 months.

**Fig. 1:**
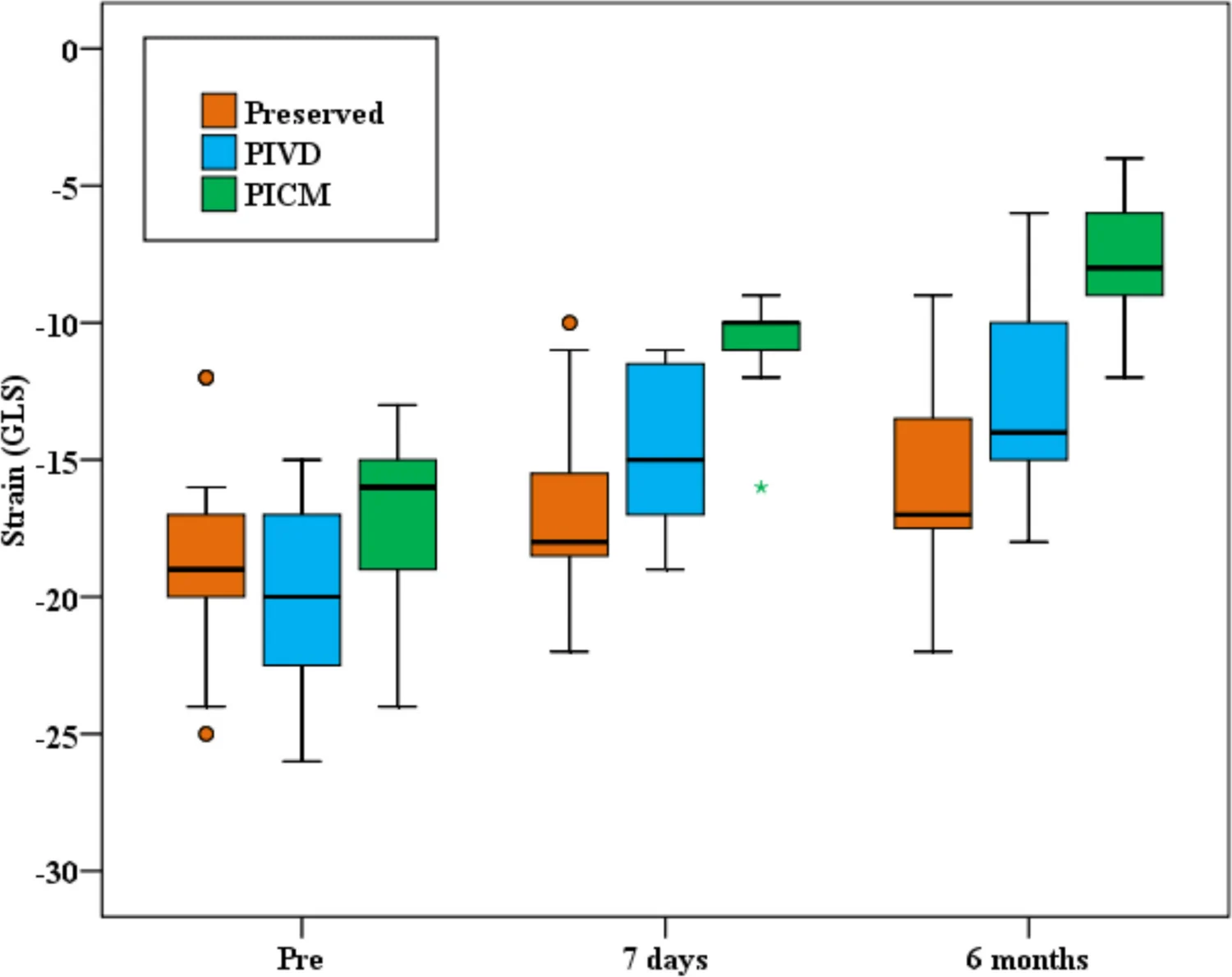
Comparison between the three studied subgroups according to LV global longitudinal strain (GLS)

**Fig. 2:**
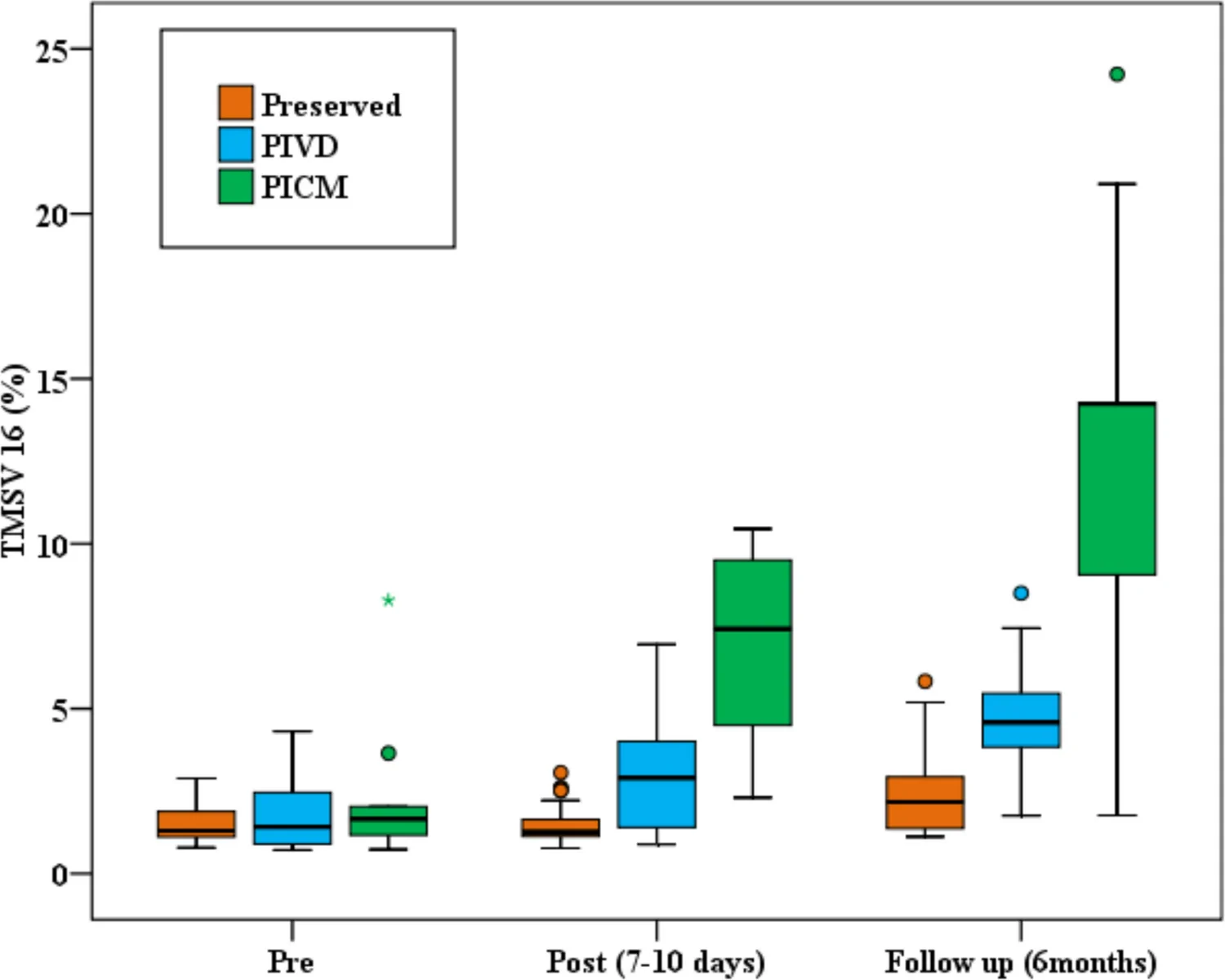
Comparison between the three studied subgroups according to TMSV 16 (%)

Similarly, the Tmsv 16-SD was comparable at baseline but increased significantly in the Dysfunction group at 7–10 days (4.50 ± 2.99% vs. 1.49 ± 0.59%, *p* < 0.001) and further worsened at 6 months (7.87 ± 5.95% vs. 2.35 ± 1.21%, *p* < 0.001). The average excursion parameter also showed a significant decline in the Dysfunction group over time. The three-subgroup analysis (Table 3) confirmed a stepwise deterioration in GLS (Fig. 1) and average excursion as well as a stepwise increase in SDI from Preserved to PIVD to PICM at all post-procedural time points (Fig. 2 and Supplementary Fig. B).

**Table 3:** Comparison between the three studied subgroups (Preserved, PIVD, PICM) according to LV global longitudinal strain (GLS) and real-time 3D SDI (Tmsv 16% and average excursion)

	Preserved (*n* = 35)	PIVD (*n* = 16)	PICM (*n* = 9)	*p*	Sig. bet. grps
LV GLS					
Pre					
Mean ± SD	− 18.66 ± 3.42	− 20.0 ± 3.39	− 17.0 ± 3.43	0.094	–
Post (7–10 days)					*p*_1_ = 0.056
Mean ± SD	− 16.69 ± 3.18	− 14.63 ± 2.90	− 10.78 ± 2.17	< 0.001^*^	*p*_2_ < 0.001^*^ *p*_3_ = 0.014^*^
% change in 7 days					*p*_1_ < 0.001^*^
Mean ± SD	10.46 ± 8.42	26.94 ± 10.22	36.11 ± 8.24	< 0.001^*^	*p*_2_ < 0.001^*^ *p*_3_ = 0.195
Follow-up (6 months)					*p*_1_ = 0.006^*^
Mean ± SD	− 15.57 ± 3.03	− 12.63 ± 3.24	− 7.89 ± 2.89	< 0.001^*^	*p*_2_ < 0.001^*^ *p*_3_ = 0.030^*^
% change in 6 months					*p*_1_ < 0.001^*^
Mean ± SD	15.91 ± 10.56	35.69 ± 10.59	49.78 ± 18.55	< 0.001^*^	*p*_2_ < 0.001^*^ *p*_3_ = 0.251
Tmsv 16–SD					
Pre					
Mean ± SD	1.48 ± 0.53	1.68 ± 1.0	2.42 ± 2.36	0.763	–
Post (7–10 days)					*p*_1_ = 0.006^*^
Mean ± SD	1.49 ± 0.59	3.03 ± 1.82	7.12 ± 2.91	< 0.001^*^	*p*_2_ < 0.001^*^ *p*_3_ = 0.018^*^
Follow-up (6 months)					*p*_1_ < 0.001^*^
Mean ± SD	2.35 ± 1.21	4.70 ± 1.80	13.50 ± 6.66	< 0.001^*^	*p*_2_ < 0.001^*^ *p*_3_ = 0.102
Average excursion					
Pre					
Mean ± SD	6.34 ± 2.48	5.94 ± 2.98	4.58 ± 3.54	0.173	–
Post (7–10 days)					*p*_1_ = 0.035^*^
Mean ± SD	6.45 ± 2.12	4.84 ± 2.27	3.12 ± 2.84	0.001^*^	*p*_2_ < 0.001^*^ *p*_3_ = 0.103
Follow-up (6 months)					*p*_1_ = 0.003^*^
Mean ± SD	5.81 ± 2.26	3.74 ± 1.86	2.34 ± 2.24	< 0.001^*^	*p*_2_ < 0.001^*^ *p*_3_ = 0.181

*H*: *H* for Kruskal–Wallis test, pairwise comparison between each 2 groups was done using post hoc test (Dunn’s for multiple comparisons test)

*IQR* inter quartile range, *SD* standard deviation, *p p-*value for comparing between the three studied subgroups, *p*_1_ *p-*value for comparing between preserved and PIVD, *p*_2_ *p-*value for comparing between preserved and PICM, *p*_3_ *p-*value for comparing between PIVD and PICM

^*^Statistically significant at *p* ≤ 0.05

### Predictors of left ventricular dysfunction

The univariate logistic regression analysis (Table 4) identifies four parameters measured at 7–10 days post-implantation as significant predictors of subsequent LV dysfunction at 6 months: ESV, GLS, Tmsv 16-SD, and Average Excursion. In the multivariate model, only GLS (OR, 1.389; 95% CI, 1.015–1.899; *p* = 0.040) and Tmsv 16-SD (OR, 5.381; 95% CI, 1.564–18.520; *p* = 0.008) remained independent predictors.

**Table 4:** Univariate and Multivariate Logistic regression analysis for the different parameters to predict Dysfunction (*n* = 25 vs. 35)

	Univariate		^#^Multivariate	
	*p*	OR (LL–UL 95%CI)	*p*	OR (LL–UL 95%CI)
ESV post (7–10 days)	0.005^*^	1.081 (1.024–1.141)	0.674	0.980 (0.892–1.077)
Strain (GLS) [7 days]	0.001^*^	1.366 (1.142–1.634)	0.040^*^	1.389 (1.015–1.899)
Tmsv 16-SD (%) post	0.001^*^	3.905 (1.777–8.584)	0.008^*^	5.381 (1.564–18.520)
Excursion average post	0.004^*^	0.589 (0.410–0.847)	0.100	0.693 (0.448–1.073)

Hosmer and Lemeshow test (*χ*^2^ = 3.987; *p* = 0.858)

*OR* odds ratio, *CI* confidence interval, *LL* lower limit, *UL* upper Limit

^#^All variables with *p* < 0.05 was included in the multivariate

^*^Statistically significant at *p* ≤ 0.05

Receiver operating characteristic (ROC) curve analysis was performed for these two independent predictors (Supplementary Table D and Fig. C). The Tmsv 16-SD demonstrated excellent predictive accuracy with an Area Under the Curve (AUC) of 0.843 (*p* < 0.001). A cut-off value of > 1.49% yielded a sensitivity of 84.0% and a specificity of 71.4%. GLS also showed good predictive value (AUC = 0.774, *p* < 0.001), with a cut-off of > − 17% providing a sensitivity of 80.0% and a specificity of 62.9%.

### Correlates of ventricular dyssynchrony

Correlation analysis (Supplementary Table E and Fig. D) revealed that in the Dysfunction group, a higher Tmsv 16-SD (greater dyssynchrony) at 7–10 days and 6 months was strongly correlated with larger ESV (*r*_s_ = 0.591, *p* = 0.002; *r*_s_ = 0.655, *p* < 0.001) and lower EF% (*r*_s_ = − 0.891, *p* < 0.001; *r*_s_ = − 0.730, *p* < 0.001). No significant correlation was found between Tmsv 16-SD and ventricular pacing percentage or lead location in either group (Supplementary Table F).

## Discussion

This prospective study, while limited by a modest sample size, provides hypothesis-generating evidence that mechanical dyssynchrony (Tmsv 16-SD) and impaired myocardial deformation (GLS), measurable within one week of right ventricular pacing, may identify patients at higher risk for subsequent LV dysfunction at 6 months. Our work adds to the growing body of evidence on pacing-induced cardiomyopathy (PICM) by highlighting the paramount importance of early mechanical alterations, which appear to precede and predict the decline in conventional volumetric measures like ejection fraction (EF).

### The evolving definition of pacing-induced ventricular dysfunction

The definition of PICM has undergone significant evolution since it was first described. Initially, it was recognized as a late complication, often defined by a dramatic decline in LVEF, typically to below 40–45%, in the absence of other clear causes [4, 5]. This classic definition, while specific, risks overlooking a larger cohort of patients who develop significant but subtler deterioration; a condition now often termed pacing-induced ventricular dysfunction (PIVD). Our study intentionally captures this spectrum by classifying dysfunction into two subgroups: PIVD (EF decline without falling below 50%) and the more classic PICM (EF < 50%). This approach is in agreement with contemporary studies that acknowledge PICM as a continuum rather than a binary outcome [10, 16]. By including PIVD, we identify patients at an earlier stage of the disease process, which is crucial for implementing preventive strategies before severe, potentially irreversible damage occurs. Our data show a clear gradient of worsening parameters from Preserved to PIVD to PICM, validating this spectrum-based model and suggesting that the deleterious effects of RV pacing start very early.

### Predictors of PICM: moving beyond traditional risk factors

Previous research has identified several baseline clinical and electrical parameters as risk factors for PICM. These include a pre-existing low-normal LVEF [17], a wide native QRS complex [6], a high percentage of ventricular pacing [7], and the presence of coronary artery disease [9, 18]. While our findings are in partial agreement with some of these associations, we observed a non-significant trend toward higher pacing burden in the Dysfunction group. Although our results showed a numerically higher mean pacing burden in the Dysfunction group compared to the Preserved group (97.3% vs. 81.3% at 6 months), this difference did not reach statistical significance (*p* = 0.078). This observation must be interpreted with considerable caution for several reasons. First, our inclusion criteria required a minimum ventricular pacing percentage of ≥ 20%, but the actual observed pacing burden was substantially higher in both groups (mean > 80%), creating a restricted range that reduces statistical power to detect associations. Second, with only 25 patients in the Dysfunction group, our study is underpowered for this analysis. Third, the definition of high pacing burden in the literature varies considerably, with some studies using thresholds of > 40% [17], others > 80% [19], and still others analyze pacing percentage as a continuous variable [20].

Therefore, a key point of divergence and strength of our study is the demonstration that these traditional factors were not significantly different between our groups at baseline. This lack of discriminatory power for baseline characteristics highlights a critical clinical challenge: relying solely on pre-implantation factors is insufficient to predict which individual patient will develop dysfunction. A patient with a normal EF and a narrow QRS can still develop PICM, as evidenced by our cohort. This is where our work suggests a new prognostic tool. We provide preliminary evidence that the *functional response* of the myocardium to pacing, quantified by GLS and SDI within the first week, is a more sensitive and earlier indicator of future risk. Our finding that SDI is a potential predictor aligns with the broader utility of this index. For instance, Bhambhani and colleagues [21], who used 3D echocardiography to demonstrate that significant mechanical dyssynchrony, quantified by SDI, is present in over 50% of heart failure patients, even those with a narrow QRS complex. Their observation that the spatial distribution of dyssynchrony is often scattered in such patients underscores that electrical surrogates like QRS duration can be blind to meaningful mechanical disturbances. Our findings suggest a parallel in the pacing population: a significant subset of patients, despite having “normal” traditional parameters, exhibits early post-pacing dyssynchrony that places them on a path toward dysfunction.

Tani and colleagues [22] demonstrated that 3D echocardiography-derived SDI is a useful parameter to assess global LV systolic dyssynchrony, showing a significant inverse correlation with LVEF (*r* = − 0.686) and a positive correlation with QRS duration (*r* = 0.407). Furthermore, they established its value in a different context, predicting response to cardiac resynchronization therapy (CRT), where an SDI > 11.9% predicted CRT response with high sensitivity and specificity. Our study extends this concept by showing that SDI can predict the onset of dysfunction caused by pacing itself, not just the response to its correction.

• **Point of agreement and advancement with GLS**

Our finding that GLS deteriorates rapidly after pacing and is a strong predictor of dysfunction is consistent with several recent studies [12, 23]. For instance, a study by Leitman et al. [23] found that GLS was significantly worse in patients who developed PICM. Our work strengthens this evidence by showing that this change is detectable extremely early (7–10 days) and provides robust prognostic value (AUC 0.774), independent of other factors. This suggests that GLS captures the immediate, subtle contractile impairment induced by dyssynchronous activation before it translates into EF reduction. Another study by Tatsumi et al. [24], using a three-dimensional speckle tracking system to derive a strain dyssynchrony index, showed that this measure could powerfully predict reverse remodeling after CRT, correlating significantly with the reduction in LV end-systolic volume (*r* = 0.80, *p* < 0.001). Their work highlights that indices combining dyssynchrony with residual myocardial contractility (like strain) offer profound clinical insight. While their study focused on predicting CRT response, the underlying principle is directly applicable to our work: the quantification of dyssynchrony and deformation provides a window into the myocardial substrate that is far more nuanced than traditional parameters.

• **Point of major contribution with the systolic dyssynchrony index (Tmsv 16-SD)**

The most powerful predictor in our multivariate model was the Tmsv 16-SD, a real-time 3D echocardiographic measure of intraventricular dyssynchrony. While the role of dyssynchrony in PICM pathophysiology is well-established [3], its quantitative assessment has often been limited by 2D techniques. Our use of 3D SDI provides a more comprehensive and reproducible assessment. The strong correlation we found between early post-pacing SDI and subsequent ESV increase and EF reduction underscores that mechanical dyssynchrony is not just a correlate but likely a primary driver of adverse remodeling. The excellent predictive accuracy of an SDI cut-off of > 1.49% (AUC 0.843) suggests it could be a valuable tool for early risk stratification.

• This pathophysiological concept is strongly supported by the foundational work of Tops et al. [3], who established the direct link between RV pacing, immediate mechanical dyssynchrony, and subsequent LV dysfunction. Our findings on the acute induction of mechanical dyssynchrony are consistent with the foundational work of Fang et al. [25], who first used real-time 3D echocardiography to quantify the high prevalence of LV systolic dyssynchrony immediately after RV apical pacing in patients with normal baseline EF.
• However, our study critically extends these observations. While Fang et al. descriptively characterized the acute phenomenon, we demonstrate that the magnitude of this early dyssynchrony is not merely an epiphenomenon but a powerful prognostic biomarker. We transition the application of RT3DE from describing the pathophysiology to enabling early, personalized risk prediction.

### Potential implications and future directions

The early timing of our predictive measurements (7–10 days post-pacing) is a key clinical advantage. This “window of opportunity” allows for proactive management in high-risk patients identified by GLS and SDI before significant irreversible remodeling sets in. One might hypothesize that interventions could include optimizing medical therapy (e.g., intensifying renin–angiotensin–aldosterone system blockade), reassessing the need for high pacing percentages, or considering early upgrade to biventricular or conductive-system pacing.

Furthermore, our correlation analysis provides insight into pathophysiology. The strong inverse relationship between SDI and EF, and its positive correlation with ESV, paints a clear picture: greater immediate dyssynchrony leads to inefficient contraction, increased energy expenditure, and ultimately, remodeling and dilatation of the LV. The absence of a significant correlation between SDI and lead location or pacing percentage within the dysfunction group, while requiring very cautious interpretation, raises an intriguing hypothesis: the mechanical response to RV pacing may be substantially heterogeneous and patient-specific, not solely determined by the site or cumulative burden of pacing. This observation aligns with the well-recognized clinical phenomenon that some patients with high-burden apical pacing maintain normal LV function for decades, while others develop PICM despite seemingly ‘favorable’ lead positions and only moderate pacing burden.

Several important methodological caveats must be emphasized when interpreting the lack of association with lead position. First, lead location was determined by standard fluoroscopic guidance in two orthogonal views, which is now recognized as an imprecise method for confirming true septal positioning. Burri and colleagues [26] demonstrated that when only standard fluoroscopic views (PA and LAO) were used, only 45% of leads intended to be septal were actually positioned on the septum by echocardiographic validation; the majority were on the anterior free wall. This misclassification bias would systematically dilute any true differences between apical and septal pacing, biasing our analysis toward the null hypothesis. Second, the largest randomized trial to address this question, the UKPACE trial [27], similarly found no difference in mortality, heart failure hospitalization, or LV function between apical and non-apical pacing over a mean follow-up of 4.6 years. Our findings are therefore consistent with the highest-quality evidence available, which suggests that simply targeting the septum under fluoroscopic guidance does not reliably prevent pacing-induced dysfunction. Third, with only 25 patients in the dysfunction group and only 7 non-apical lead placements, our study was profoundly underpowered for this subgroup analysis; the absence of statistical significance may well represent a Type II error rather than a true biological null effect.

### 3D echocardiography for dyssynchrony assessment: required or complementary?

An important practical question is whether three-dimensional echocardiography is strictly necessary for dyssynchrony assessment or whether two-dimensional methods might suffice. Vaidya and colleagues [28] directly compared 2D-strain-derived and 3D-echocardiographic parameters, reporting excellent correlation for LVEF and volumes but, critically, no significant correlation between 2D-strain-derived and 3D-derived systolic dyssynchrony indices (*r* = 0.171). This finding has important implications: while 2D echocardiography may adequately assess LVEF and volumes in paced patients, the quantification of mechanical dyssynchrony appears to require 3D acquisition. The 2D methods assess dyssynchrony in single imaging planes, whereas 3DE captures the entire LV volume and provides a global measure of temporal contraction disparity across all segments simultaneously. Therefore, while we acknowledge that 3DE is not universally available and requires specific expertise, our data suggest that if the goal is to quantify global LV mechanical dyssynchrony, 3D echocardiography may be uniquely suited for this purpose. Two-dimensional echocardiography remains entirely appropriate for routine assessment of LV volumes and ejection fraction in paced patients. However, for dyssynchrony quantification, 2D methods appear to capture a different aspect of myocardial mechanics and cannot be considered interchangeable with 3D-derived SDI. Whether 2D surrogates can be refined to approximate 3D SDI remains an important area for future investigation.

### Relevance of conduction system pacing

Our study must be interpreted in the context of rapidly evolving pacing technologies. Left bundle branch area pacing (LBBAP) has emerged as a promising alternative to conventional RV pacing, achieving physiological ventricular activation via the native conduction system with narrow-paced QRS duration and preserved ventricular synchrony. Recent comparative studies have demonstrated a significantly lower PICM incidence with LBBAP (4.3%) compared to RV septal pacing (24%) in patients with high pacing burden [29]. Furthermore, among patients who have already developed PICM from chronic RV pacing, upgrading to LBBAP may be superior to biventricular pacing for LVEF recovery (9.6% vs. 4.9% absolute improvement at 6 months, *p* = 0.008) [30].

These emerging data raise the question: if LBBAP can prevent PICM, is there still a role for early risk stratification with SDI and GLS? We propose that these approaches are complementary rather than competitive. First, LBBAP is not universally feasible or successful; implantation failure rates and the need for lead revision are not negligible, and many patients continue to receive conventional RV pacing systems. Second, even with LBBAP, some patients develop residual dyssynchrony and may benefit from early identification. Third, for the large prevalent population of patients with existing RV pacing systems, risk stratification remains highly relevant to guide decisions about upgrade procedures. Thus, while we enthusiastically support the adoption of physiological pacing when feasible, we believe that early post-pacing mechanical assessment retains clinical relevance in the current era.

### Study limitations

Our study has several limitations. Most importantly, the modest sample size (*n* = 60) significantly limits the robustness of our findings and precludes definitive conclusions. While we observed strong associations between early SDI/GLS and subsequent dysfunction, the relatively small number of events (25 dysfunction cases, including only 9 PICM cases) increases the risk of both false-positive and false-negative findings. The proposed cut-off values (> 1.49% for SDI, > − 17% for GLS) should be considered preliminary and require independent validation in larger, preferably multicenter, cohorts before any clinical application can be considered. Our study is best viewed as hypothesis-generating, providing rationale for larger prospective investigations rather than evidence for immediate clinical implementation. Second, the follow-up period was 6 months; longer-term follow-up would be valuable to assess the durability of these predictions and the natural history of PIVD. Third, three-dimensional echocardiography is highly dependent on adequate acoustic windows and image quality. Suboptimal visualization of the endocardial border, particularly in the lateral and anterior walls, can compromise the accuracy of semi-automated border detection and the derived SDI values. Although we excluded patients with poor echocardiographic windows at enrollment, this introduces selection bias and limits the generalizability of our findings to the broader pacemaker population, which includes many patients with suboptimal acoustic windows. Furthermore, the expertise required for 3D acquisition and offline analysis may not be available in all echocardiography laboratories, potentially limiting the widespread applicability of this technique. Finally, our inclusion criterion of ≥ 20% ventricular pacing, combined with the very high observed pacing burden in the majority of patients (mean > 80%), resulted in a restricted range that limited our ability to detect associations between pacing percentage and outcomes.

### Conclusion

In conclusion, this hypothesis-generating study suggests that the development of pacing-induced ventricular dysfunction may be detectable very early after pacemaker implantation through assessment of mechanical dyssynchrony (3D SDI) and myocardial deformation (GLS). These preliminary findings, if confirmed in larger prospective cohorts, could inform the design of interventional trials aimed at preventing progression to overt cardiomyopathy. For the present, our results should be considered exploratory and require independent validation.

## Supplementary Information

Below is the link to the electronic supplementary material.

ESM 1 (DOCX 1.45 MB)

ESM 2 (DOCX 46.1 KB)

## Data Availability

The data that support the findings of this study are not publicly available because they contain information that could compromise the privacy of research participants. Data are available from the corresponding author upon reasonable request.

## Declarations

### Pre-registered clinical trial number

This was an observational study, not a clinical research, where patients would be subjected to a new therapy. For this reason, we did not register the study.

## Abbreviations

*2D*: Two-dimensional
*3D*: Three-dimensional
*3DE*: Three-dimensional echocardiography
*AF*: Atrial fibrillation
*AV*: Atrioventricular
*AUC*: Area under the curve
*CI*: Confidence interval
*COP*: Cardiac output
*CRT*: Cardiac resynchronization therapy
*DDD*: Dual-chamber pacing mode
*ECG*: Electrocardiogram
*EDV*: End-diastolic volume
*EF*: Ejection fraction
*ESV*: End-systolic volume
*GLS*: Global longitudinal strain
*IQR*: Interquartile range
*LA*: Left atrium
*LBBAP*: Left bundle branch area pacing
*LBBB*: Left bundle branch block
*LV*: Left ventricular/left ventricle
*LVEF*: Left ventricular ejection fraction
*MRI*: Magnetic resonance imaging
*NPV*: Negative predictive value
*OR*: Odds ratio
*PICM*: Pacing-induced cardiomyopathy
*PIVD*: Pacing-induced ventricular dysfunction
*PPV*: Positive predictive value
*RAAS*: Renin–angiotensin–aldosterone system
*ROC*: Receiver operating characteristic
*RT3DE*: Real-time three-dimensional echocardiography
*RVA*: Right ventricular apex
*RV*: Right ventricular/right ventricle
*RVS*: Right ventricular septum
*SD*: Standard deviation
*SDI*: Systolic dyssynchrony index
*SPSS*: Statistical package for the social sciences
*SV*: Stroke volume
*Tmsv*: Time to minimum systolic volume
*Tmsv 16-SD*: Standard deviation of time to minimum systolic volume for 16 segments
*VVI*: Single-chamber ventricular pacing mode
