# Supplementary material for: Real-time three-dimensional systolic dyssynchrony index predicts pacing-induced left ventricular dysfunction and cardiomyopathy
Source: Clin Res Cardiol. 2026 Apr 27;115(12):2172–85. doi: 10.1007/s00392-026-02921-7 (PMC13751685; doi:10.1007/s00392-026-02921-7)
Supplement: Supplementary file 1 [file 392_2026_2921_MOESM1_ESM.docx]

**Figure (A):** Flowchart of patient selection process and outcomes. Of 108 patients assessed, 42 were excluded prior to implantation. Sixty-six patients underwent PPM implantation, of which 6 were lost during follow-up, resulting in a final analytic cohort of 60 patients. Outcomes: Preserved LV function (58.3%), Pacing-Induced Ventricular Dysfunction - PIVD (31.7%), and Pacing-Induced Cardiomyopathy - PICM (10.0%). LVEF, left ventricular ejection fraction; FU, follow-up; PPM, Permanent Pacemaker; MI, Myocardial Infarction; LVF, Left Ventricular Function.

**Figure (B):	Comparison between the three studied subgroups according to average Excursion**

**Figure (C):	ROC curve for Strain (GLS) [7-10 days] and Tmsv 16-SD (%) [7-10 days] to predict Dysfunction (n = 25 vs. 35)**

**Figure (D):	Correlation between Tmsv 16-SD (%) in the Dysfunction group (n = 25) with: (A) ESV at 7-10 days (B) ESV at 6 months (C) EF% at 7-10 days (D) EF% at 6 months**.
