# Supplementary material for: Real-time three-dimensional systolic dyssynchrony index predicts pacing-induced left ventricular dysfunction and cardiomyopathy
Source: Clin Res Cardiol. 2026 Apr 27;115(12):2172–85. doi: 10.1007/s00392-026-02921-7 (PMC13751685; doi:10.1007/s00392-026-02921-7)
Supplement: Supplementary file 2 [file 392_2026_2921_MOESM2_ESM.docx]

**Table (A):	Comparison between the two studied groups (Preserved, Dysfunction) according to demographic, ECG, and device characteristics.**

|  | **Total (n = 60)** | **Preserved (n = 35)** | **Dysfunction (n = 25)** | **p** |
| --- | --- | --- | --- | --- |
| **Sex** |  |  |  |  |
| Male | 32 (53.3%) | 19 (54.3%) | 13 (52.0%) | 0.861 |
| Female | 28 (46.7%) | 16 (45.7%) | 12 (48.0%) |  |
| **Age (years)** |  |  |  |  |
| Mean ± SD. | 62.43 ± 16.34 | 63.11 ± 18.99 | 61.48 ± 11.99 | 0.706 |
| **DM** | 15 (25.0%) | 10 (28.6%) | 5 (20.0%) | 0.450 |
| **HTN** | 24 (40.0%) | 15 (42.9%) | 9 (36.0%) | 0.593 |
| **IHD** | 1 (1.7%) | 0 (0.0%) | 1 (4.0%) | ^FE^p=0.417 |
| **Heart rate** |  |  |  |  |
| **Pre** |  |  |  |  |
| Mean ± SD. | 42.05 ± 8.97 | 43.20 ± 10.26 | 40.44 ± 6.61 | 0.489 |
| **Post (7-10 days)** |  |  |  |  |
| Mean ± SD. | 70.37 ± 12.68 | 70.83 ± 12.74 | 69.72 ± 12.83 | 0.704 |
| **Follow up (6months)** |  |  |  |  |
| Mean ± SD. | 68.55 ± 10.75 | 68.40 ± 10.55 | 68.76 ± 11.24 | 0.897 |
| **Native QRS Width**  Mean ± SD. | 119.8 ± 33.58 | 115.1 ± 31.93 | 126.4 ± 35.37 | 0.203 |
| **Paced QRS Width** |  |  |  |  |
| Mean ± SD. | 153.2 ± 22.49 | 153.9 ± 22.03 | 152.2 ± 23.54 | 0.781 |
| **Paced QRS Axis** |  |  |  |  |
| No | 9 (15.0%) | 6 (17.1%) | 3 (12.0%) | ^MC^p= 0.712 |
| Right | 3 (5.0%) | 1 (2.9%) | 2 (8.0%) |  |
| Ex. Right | 1 (1.7%) | 1 (2.9%) | 0 (0.0%) |  |
| Left | 47 (78.3%) | 27 (77.1%) | 20 (80.0%) |  |
| **Device type** |  |  |  |  |
| VVI | 32 (53.3%) | 21 (60.0%) | 11 (44.0%) | 0.221 |
| DDD | 28 (46.7%) | 14 (40.0%) | 14 (56.0%) |  |
| **Lead site** |  |  |  |  |
| RVA | 48 (80.0%) | 27 (77.1%) | 21 (84.0%) | ^MC^p= 0.641 |
| RVS | 7 (11.7%) | 4 (11.4%) | 3 (12.0%) |  |
| RVFW | 5 (8.3%) | 4 (11.4%) | 1 (4.0%) |  |
| **V. pacing (%) [7 days]** |  |  |  |  |
| Mean ± SD. | 82.08 ± 27.31 | 77.81 ± 27.50 | 88.05 ± 26.44 | 0.167 |
| **V. pacing (%) [5 months]** |  |  |  |  |
| Mean ± SD. | 87.97 ± 22.01 | 81.31 ± 26.46 | 97.28 ± 6.67 | 0.078 |

IQR: **Inter quartile range**	SD: **Standard deviation**

p: p value for comparing between the two studied groups

**Table (B):	Comparison between the two studied groups (Preserved, Dysfunction) according to 3D Echo derived LV volumes, ejection fraction, stroke volume, and cardiac output.**

|  |  | | **Total (n = 60)** | | **Preserved (n = 35)** | | **Dysfunction (n = 25)** | | **p** | |  |
| --- | --- | --- | --- | --- | --- | --- | --- | --- | --- | --- | --- |
| **EDV** | **Pre** | |  | |  | |  | |  | |  |
|  | Mean ± SD. | | 104.4 ± 24.15 | | 103.2 ± 27.75 | | 106.2 ± 18.37 | | 0.609 | |  |
|  | **Post (7-10 days)** | |  | |  | |  | |  | |  |
|  | Mean ± SD. | | 88.43 ± 22.13 | | 84.86 ± 24.12 | | 93.44 ± 18.31 | | 0.140 | |  |
|  | **Follow up (6months)** | |  | |  | |  | |  | |  |
|  | Mean ± SD. | | 91.28 ± 25.46 | | 88.69 ± 24.50 | | 94.92 ± 26.82 | | 0.354 | |  |
| **ESV** | **Pre** | |  | |  | |  | |  | |  |
|  | Mean ± SD. | | 31.43 ± 10.82 | | 31.86 ± 12.05 | | 30.84 ± 9.01 | | 0.710 | |  |
|  | **Post (7-10 days)** | |  | |  | |  | |  | |  |
|  | Mean ± SD. | | 32.15 ± 11.73 | | 28.31 ± 11.51 | | 37.52 ± 9.96 | | 0.002^*^ | |  |
|  | **Follow up (6months)** | |  | |  | |  | |  | |  |
|  | Mean ± SD. | | 39.28 ± 18.28 | | 33.60 ± 10.87 | | 47.24 ± 23.26 | | 0.004^*^ | |  |
| **EF%** | **Pre** | |  | |  | |  | |  | |  |
|  | Mean ± SD. | | 69.93 ± 6.75 | | 69.40 ± 6.50 | | 70.68 ± 7.15 | | 0.474 | |  |
|  | **Post (7-10 days)** | |  | |  | |  | |  | |  |
|  | Mean ± SD. | | 63.23 ± 8.62 | | 65.97 ± 6.72 | | 59.40 ± 9.60 | | 0.005^*^ | |  |
|  | **Follow up (6months)** | |  | |  | |  | |  | |  |
|  | Mean ± SD. | | 57.87 ± 10.76 | | 63.60 ± 5.04 | | 49.84 ± 11.57 | | <0.001^*^ | |  |
| **SV** | | **Pre** | |  | |  | |  | |  | |
|  |  | Mean ± SD. | | 72.68 ± 18.35 | | 71.40 ± 19.55 | | 74.48 ± 16.74 | | 0.526 | |
|  |  | **Post (7-10 days)** | |  | |  | |  | |  | |
|  |  | Mean ± SD. | | 56.23 ± 15.58 | | 56.43 ± 16.81 | | 55.96 ± 14.02 | | 0.910 | |
|  |  | **Follow up (6months)** | |  | |  | |  | |  | |
|  |  | Mean ± SD. | | 52.20 ± 15.60 | | 55.09 ± 16.18 | | 48.16 ± 14.09 | | 0.090 | |
| **COP** | | **Pre** | |  | |  | |  | |  | |
|  |  | Mean ± SD. | | 3.07 ± 0.96 | | 3.11 ± 1.08 | | 3.02 ± 0.79 | | 0.720 | |
|  |  | **Post (7-10 days)** | |  | |  | |  | |  | |
|  |  | Mean ± SD. | | 3.97 ± 1.13 | | 4.05 ± 1.18 | | 3.87 ± 1.07 | | 0.553 | |
|  |  | **Follow up (6 months)** | |  | |  | |  | |  | |
|  |  | Mean ± SD. | | 3.56 ± 1.13 | | 3.81 ± 1.22 | | 3.21 ± 0.89 | | 0.041^*^ | |

p: p value for comparing between the two studied groups	*: Statistically significant at p ≤ 0.05

**Table (C):	Comparison between the two studied groups (Preserved, Dysfunction) according to LV global longitudinal strain (GLS), real-time 3D SDI (Tmsv 16% and average excursion)**

|  | **Total (n = 60)** | **Preserved (n = 35)** | **Dysfunction (n = 25)** |  |
| --- | --- | --- | --- | --- |
| **LV GLS** |  |  |  |  |
| **Pre** |  |  |  |  |
| Mean ± SD. | -18.8 ± 3.49 | -18.7 ± 3.42 | -18.9 ± 3.64 | 0.940 |
| **Post (7-10 days)** |  |  |  |  |
| Mean ± SD. | -15.2 ± 3.60 | -16.7 ± 3.18 | -13.2 ± 3.22 | <0.001^*^ |
| **% change in 7 days** |  |  |  |  |
| Mean ± SD. | 18.70 ± 13.47 | 10.46 ± 8.42 | 30.24 ± 10.40 | <0.001^*^ |
| **Follow up (6months)** |  |  |  |  |
| Mean ± SD. | -13.6 ± 4.08 | -15.6 ± 3.03 | -10.9 ± 3.84 | <0.001^*^ |
| **% change in 6 months** |  |  |  |  |
| Mean ± SD. | 26.27 ± 17.65 | 15.91 ± 10.56 | 40.76 ± 15.25 | <0.001^*^ |
| **Tmsv 16-SD** |  |  |  |  |
| **Pre** |  |  |  |  |
| Mean ± SD. | 1.67 ± 1.13 | 1.48 ± 0.53 | 1.95 ± 1.61 | 0.805 |
| **Post (7-10 days)** |  |  |  |  |
| Mean ± SD. | 2.74 ± 2.46 | 1.49 ± 0.59 | 4.50 ± 2.99 | <0.001^*^ |
| **Follow up (6months)** |  |  |  |  |
| Mean ± SD. | 4.65 ± 4.77 | 2.35 ± 1.21 | 7.87 ± 5.95 | <0.001^*^ |
| **Average Excursion** |  |  |  |  |
| **Pre** |  |  |  |  |
| Mean ± SD. | 5.97 ± 2.81 | 6.34 ± 2.48 | 5.45 ± 3.19 | 0.277 |
| **Post (7-10 days)** |  |  |  |  |
| Mean ± SD. | 5.52 ± 2.55 | 6.45 ± 2.12 | 4.22 ± 2.57 | 0.001^*^ |
| **Follow up (6months)** |  |  |  |  |
| Mean ± SD. | 4.74 ± 2.52 | 5.81 ± 2.26 | 3.24 ± 2.08 | <0.001^*^ |

p: p value for comparing between the two studied groups	*: Statistically significant at p ≤ 0.05

**Table (D):	Prognostic performance for Strain (GLS) [7 days] and Tmsv 16-SD (%) Post to predict Dysfunction (n = 25 vs. 35)**

|  | **AUC** | **p** | **95% C.I** | **Cut off** | **Sensitivity** | **Specificity** | **PPV** | **NPV** |
| --- | --- | --- | --- | --- | --- | --- | --- | --- |
| **Strain (GLS) [7 days]** | 0.774 | <0.001^*^ | 0.653 – 0.895 | >-17^#^ | 80.0 | 62.86 | 60.6 | 81.5 |
| **Tmsv 16-SD (%) Post** | 0.843 | <0.001^*^ | 0.726 – 0.960 | >1.49 | 84.0 | 71.43 | 67.7 | 86.2 |

AUC: Area Under a Curve	p value: Probability value	CI: Confidence Intervals

NPV: Negative predictive value	PPV: Positive predictive value

*: Statistically significant at p ≤ 0.05	#Cut off was choose according to Youden index

**Table (E):	Correlation between Tmsv 16-SD (%) with different parameters in each group**

|  |  | **Tmsv 16-SD (%)** | | | | | |
| --- | --- | --- | --- | --- | --- | --- | --- |
|  |  | **Preserved (n = 35)** | | | **Dysfunction (n = 25)** | | |
|  |  | **Pre** | **Post** | **Follow up** | **Pre** | **Post** | **Follow up** |
| **QRS Width** | **r_s_** | 0.223 | 0.218 | – | 0.111 | -0.299 | – |
|  | **p** | 0.197 | 0.209 | – | 0.597 | 0.147 | – |
| **V. pacing (%)** | **r_s_** | – | 0.103 | – | – | 0.140 | – |
|  | **p** | – | 0.555 | – | – | 0.504 | – |
| **ESV** | **r_s_** | 0.271 | -0.030 | -0.160 | 0.376 | 0.591^*^ | 0.655^*^ |
|  | **p** | 0.115 | 0.863 | 0.358 | 0.064 | 0.002^*^ | <0.001^*^ |
| **EF%** | **r_s_** | -0.113 | -0.231 | -0.188 | -0.509^*^ | -0.891^*^ | -0.730^*^ |
|  | **p** | 0.519 | 0.182 | 0.280 | 0.009^*^ | <0.001^*^ | <0.001^*^ |
| **SV-3D** | **r_s_** | 0.245 | 0.080 | 0.002 | -0.233 | -0.517^*^ | -0.069 |
|  | **p** | 0.156 | 0.647 | 0.992 | 0.263 | 0.008^*^ | 0.743 |
| **Strain (GLS) [Pre]** | **r_s_** | -0.239 | -0.084 | 0.116 | 0.215 | 0.346 | 0.354 |
|  | **p** | 0.166 | 0.631 | 0.509 | 0.301 | 0.090 | 0.082 |
| **Excursion Average** | **r_s_** | -0.248 | -0.110 | -0.149 | -0.066 | -0.560^*^ | -0.175 |
|  | **p** | 0.150 | 0.531 | 0.393 | 0.754 | 0.004^*^ | 0.402 |

**r_s_: Spearman coefficient**

*: Statistically significant at p ≤ 0.05

**Table (F):	Relation between Tmsv 16-SD (%) post with device type and Lead site in each group**

|  | **Tmsv 16-SD (%)** | | | | | |
| --- | --- | --- | --- | --- | --- | --- |
|  | **Preserved (n = 35)** | | | **Dysfunction (n = 25)** | | |
|  | **No.** | **Mean ± SD.** | **Median (Min. – Max.)** | **No.** | **Mean ± SD.** | **Median (Min. – Max.)** |
| **Device type** |  |  |  |  |  |  |
| VVI | **21** | 1.53 ± 0.66 | 1.30 (0.77 – 3.06) | **11** | 3.68 ± 2.88 | 2.90 (1.08 – 10.29) |
| DDD | **14** | 1.43 ± 0.47 | 1.26 (0.78 – 2.58) | **14** | 5.14 ± 3.01 | 4.32 (0.89 – 10.45) |
| **U (p)** | U=147.00 (p=1.000) | | | U=52.00 (p=0.183) | | |
| **Lead site** |  |  |  |  |  |  |
| RVA | **27** | 1.45 ± 0.54 | 1.25 (0.78 – 2.61) | **21** | 4.30 ± 3.02 | 3.29 (0.89 – 10.45) |
| RVS | **4** | 1.47 ± 1.07 | 1.03 (0.77 – 3.06) | **3** | 5.05 ± 3.47 | 5.10 (1.56 – 8.50) |
| RVFW | **4** | 1.77 ± 0.37 | 1.75 (1.37 – 2.22) | **1** | 6.95^#^ | |
| **Test of Sig. (p)** | H=3.615 (p=0.164) | | | U=26.00 (p=0.680) | | |

SD: **Standard deviation	U: Mann Whitney test**	H: H for **Kruskal Wallis test**

p: p value for Relation between Tmsv 16-SD (%) post with device type and Lead

*: Statistically significant at p ≤ 0.05

#: Excluded from the comparison due to small number of case (n = 1)
